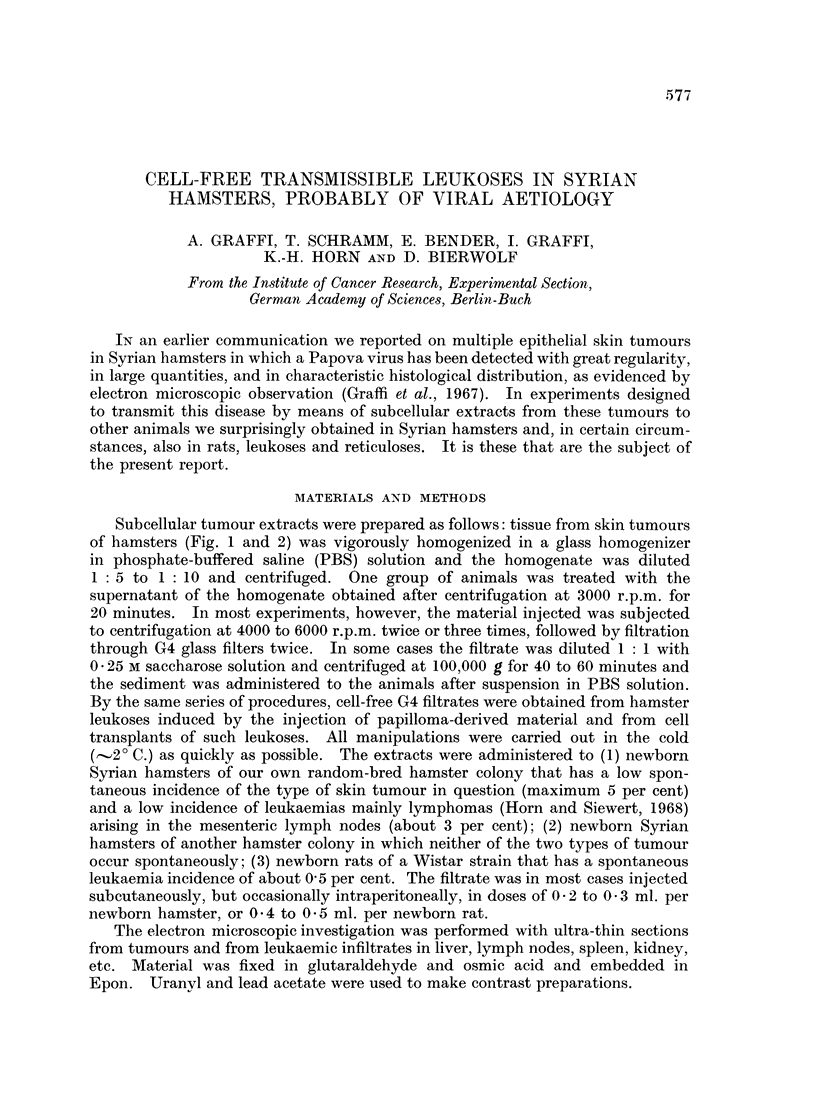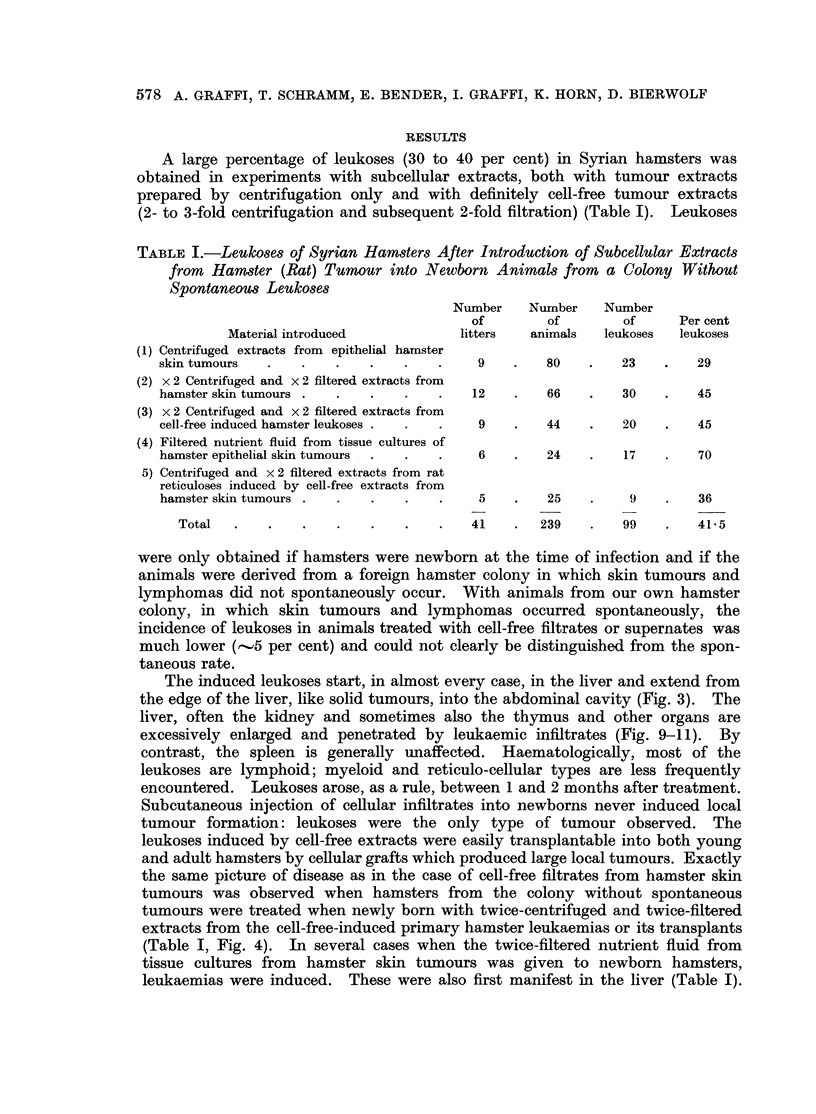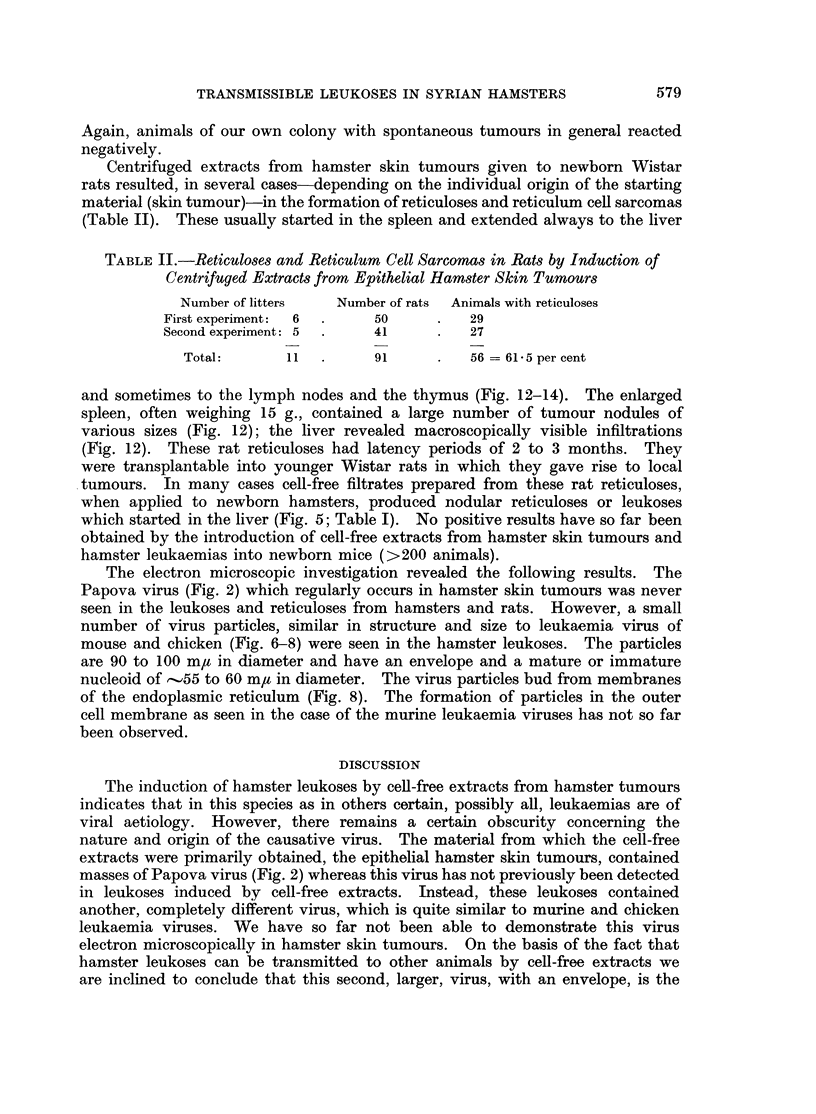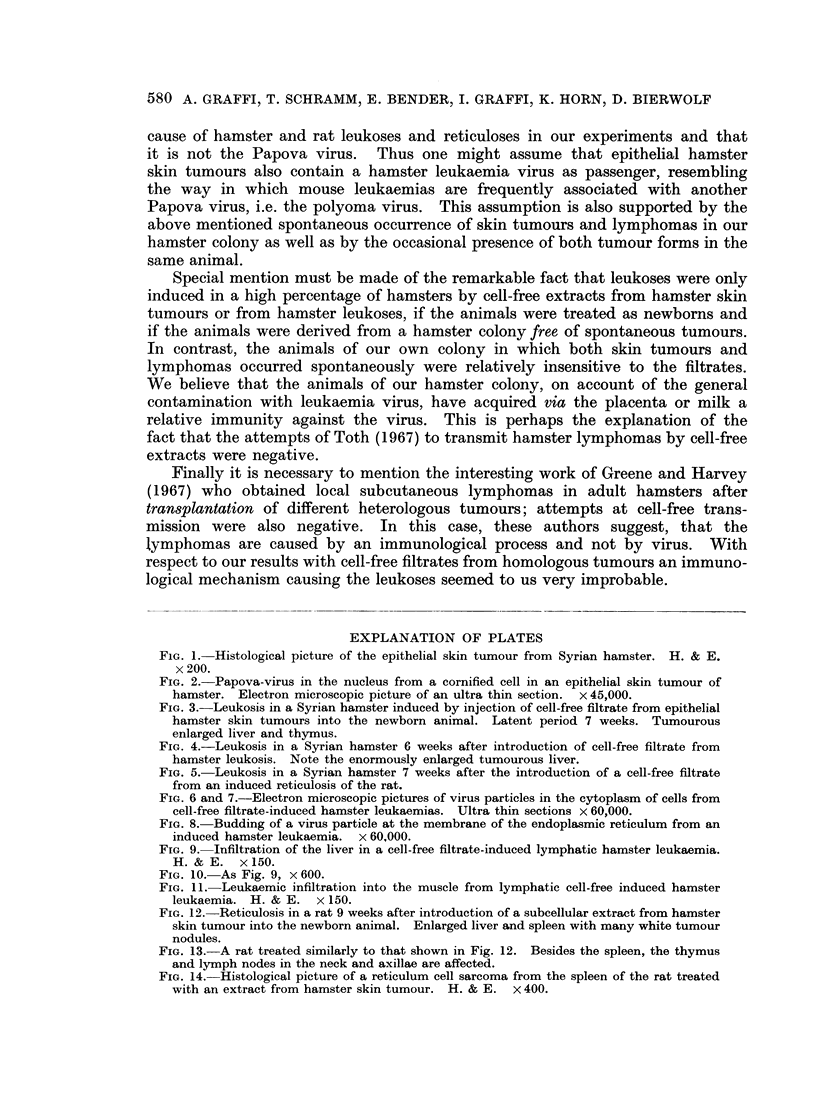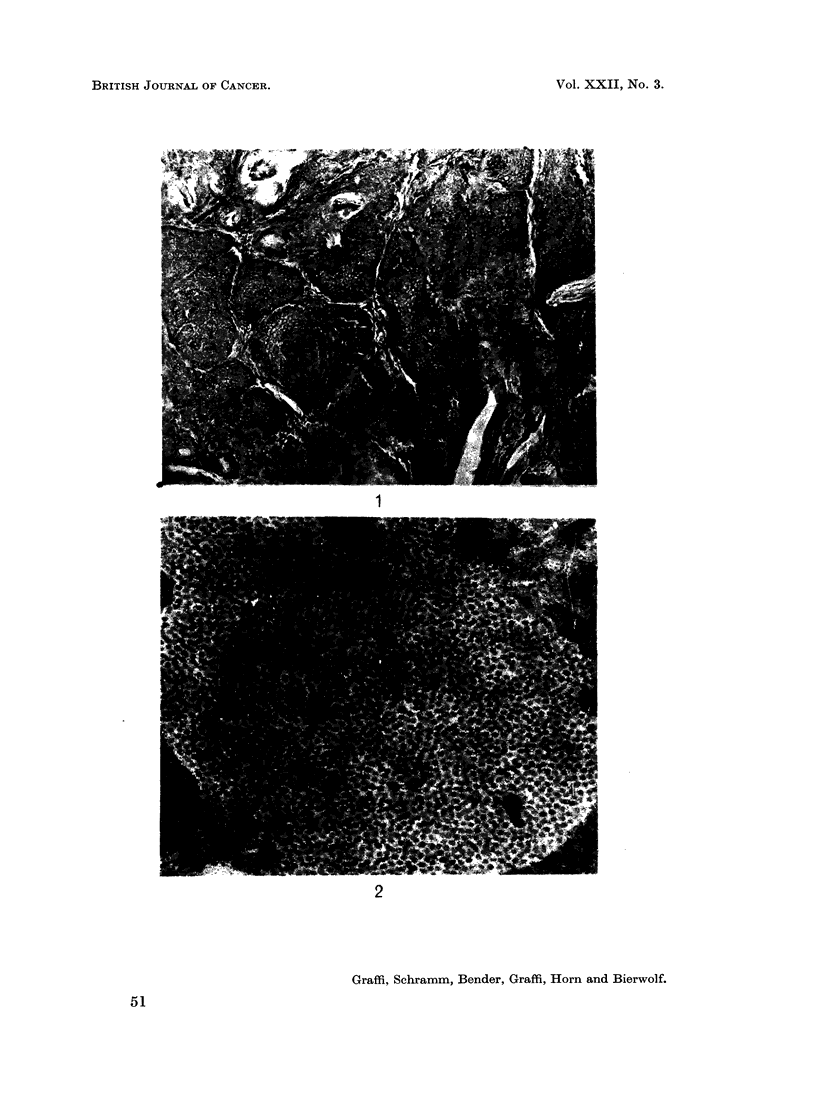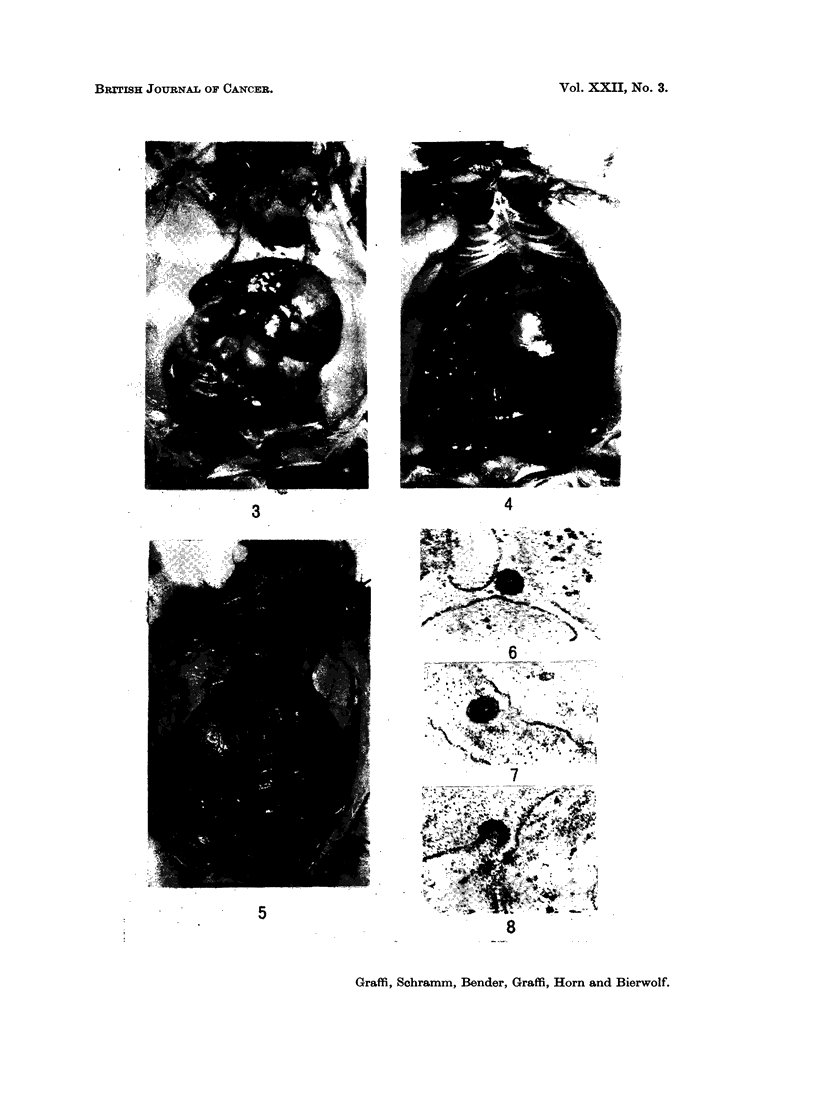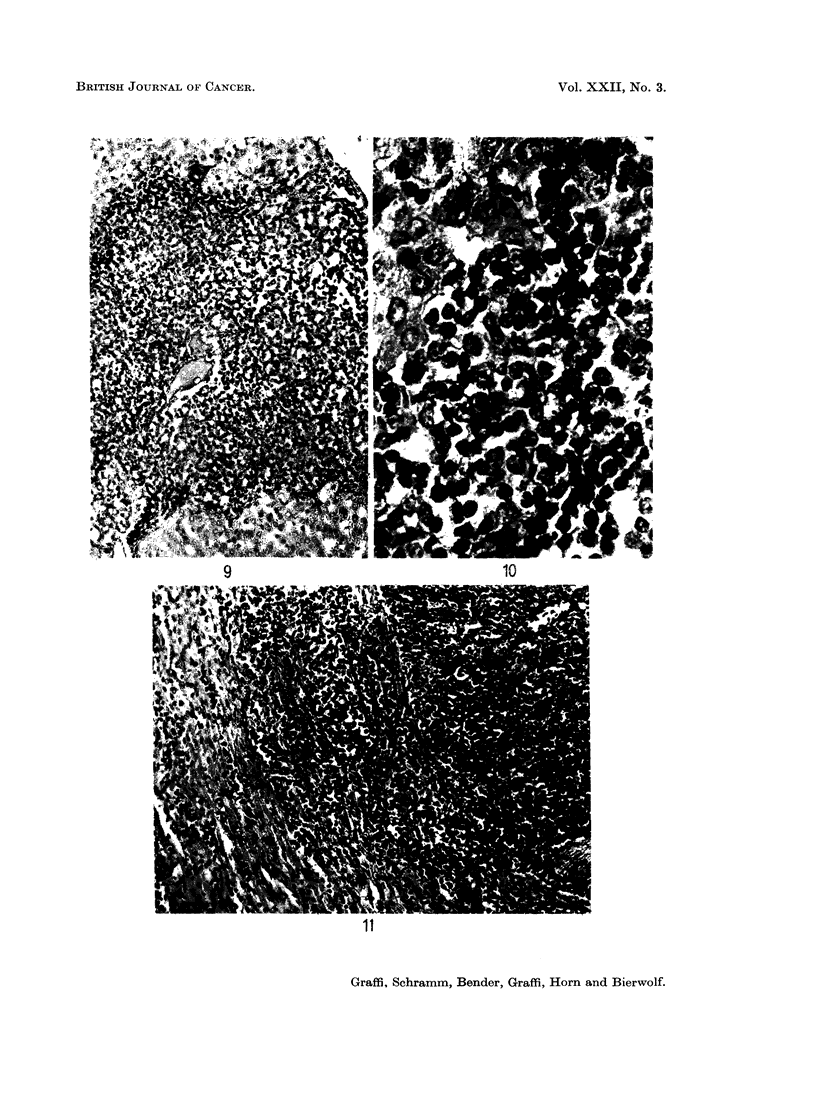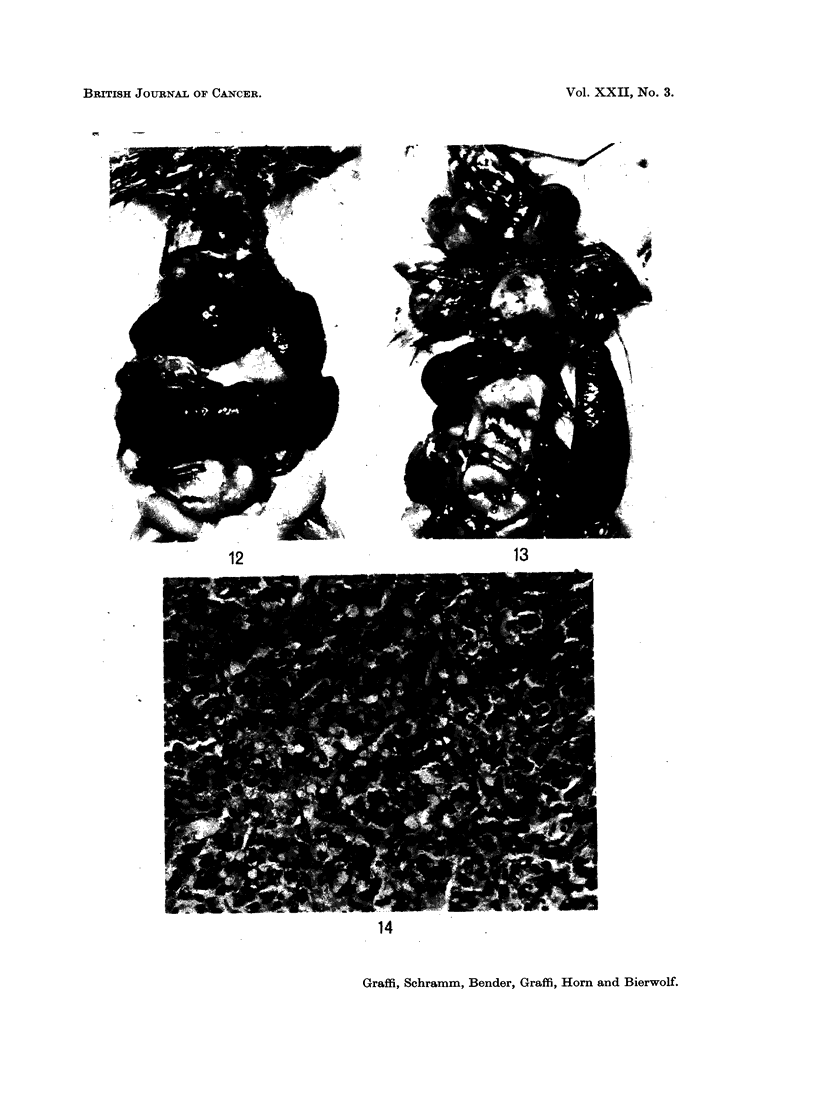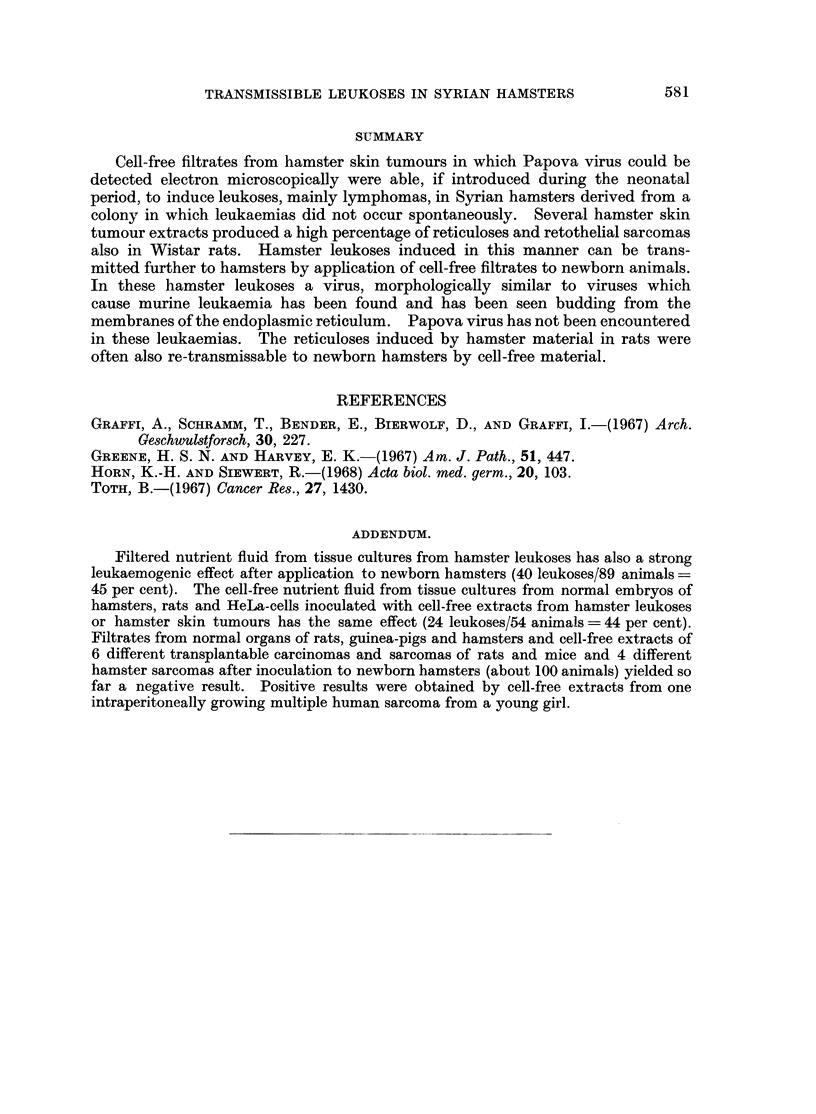# Cell-free transmissible leukoses in Syrian hamsters, probably of viral aetiology.

**DOI:** 10.1038/bjc.1968.68

**Published:** 1968-09

**Authors:** A. Graffi, T. Schramm, E. Bender, I. Graffi, K. H. Horn, D. Bierwolf

## Abstract

**Images:**


### CELL-FREE TRANSMISSIBLE LEUKOSES IN SYRIAN HAMSTERS, PROBABLY OF VIRAL AETIOLOGY

A. GRAFFI, T. SCHRAMM, E. BENDER, I. GRAFFI,
K.-H. HORN AND D. BIERWOLF

*From the Institute of Cancer Research, Experimental Section,
German Academy of Sciences, Berlin-Buch*

IN an earlier communication we reported on multiple epithelial skin tumours in Syrian hamsters in which a Papova virus has been detected with great regularity, in large quantities, and in characteristic histological distribution, as evidenced by electron microscopic observation (Graffi *et al.*, 1967). In experiments designed to transmit this disease by means of subcellular extracts from these tumours to other animals we surprisingly obtained in Syrian hamsters and, in certain circumstances, also in rats, leukoses and reticuloses. It is these that are the subject of the present report.

### MATERIALS AND METHODS

Subcellular tumour extracts were prepared as follows: tissue from skin tumours of hamsters (Fig. 1 and 2) was vigorously homogenized in a glass homogenizer in phosphate-buffered saline (PBS) solution and the homogenate was diluted 1 : 5 to 1 : 10 and centrifuged. One group of animals was treated with the supernatant of the homogenate obtained after centrifugation at 3000 r.p.m. for 20 minutes. In most experiments, however, the material injected was subjected to centrifugation at 4000 to 6000 r.p.m. twice or three times, followed by filtration through G4 glass filters twice. In some cases the filtrate was diluted 1 : 1 with 0·25 M saccharose solution and centrifuged at 100,000 *g* for 40 to 60 minutes and the sediment was administered to the animals after suspension in PBS solution. By the same series of procedures, cell-free G4 filtrates were obtained from hamster leukoses induced by the injection of papilloma-derived material and from cell transplants of such leukoses. All manipulations were carried out in the cold ($\sim$2° C.) as quickly as possible. The extracts were administered to (1) newborn Syrian hamsters of our own random-bred hamster colony that has a low spontaneous incidence of the type of skin tumour in question (maximum 5 per cent) and a low incidence of leukaemias mainly lymphomas (Horn and Siewert, 1968) arising in the mesenteric lymph nodes (about 3 per cent); (2) newborn Syrian hamsters of another hamster colony in which neither of the two types of tumour occur spontaneously; (3) newborn rats of a Wistar strain that has a spontaneous leukaemia incidence of about 0·5 per cent. The filtrate was in most cases injected subcutaneously, but occasionally intraperitoneally, in doses of 0·2 to 0·3 ml. per newborn hamster, or 0·4 to 0·5 ml. per newborn rat.

The electron microscopic investigation was performed with ultra-thin sections from tumours and from leukaemic infiltrates in liver, lymph nodes, spleen, kidney, etc. Material was fixed in glutaraldehyde and osmic acid and embedded in Epon. Uranyl and lead acetate were used to make contrast preparations.

### RESULTS

A large percentage of leukoses (30 to 40 per cent) in Syrian hamsters was obtained in experiments with subcellular extracts, both with tumour extracts prepared by centrifugation only and with definitely cell-free tumour extracts (2- to 3-fold centrifugation and subsequent 2-fold filtration) (Table I). Leukoses

TABLE I.—*Leukoses of Syrian Hamsters After Introduction of Subcellular Extracts from Hamster (Rat) Tumour into Newborn Animals from a Colony Without Spontaneous Leukoses*

| Material introduced | Number of litters | Number of animals | Number of leukoses | Per cent leukoses |
|---|---|---|---|---|
| (1) Centrifuged extracts from epithelial hamster skin tumours . . . . . . . | 9 | 80 | 23 | 29 |
| (2) ×2 Centrifuged and ×2 filtered extracts from hamster skin tumours . . . . . . | 12 | 66 | 30 | 45 |
| (3) ×2 Centrifuged and ×2 filtered extracts from cell-free induced hamster leukoses . . . . | 9 | 44 | 20 | 45 |
| (4) Filtered nutrient fluid from tissue cultures of hamster epithelial skin tumours . . . | 6 | 24 | 17 | 70 |
| 5) Centrifuged and ×2 filtered extracts from rat reticuloses induced by cell-free extracts from hamster skin tumours . . . . . . | 5 | 25 | 9 | 36 |
| Total . . . . . . . . | 41 | 239 | 99 | 41·5 |

were only obtained if hamsters were newborn at the time of infection and if the animals were derived from a foreign hamster colony in which skin tumours and lymphomas did not spontaneously occur. With animals from our own hamster colony, in which skin tumours and lymphomas occurred spontaneously, the incidence of leukoses in animals treated with cell-free filtrates or supernates was much lower (~5 per cent) and could not clearly be distinguished from the spontaneous rate.

The induced leukoses start, in almost every case, in the liver and extend from the edge of the liver, like solid tumours, into the abdominal cavity (Fig. 3). The liver, often the kidney and sometimes also the thymus and other organs are excessively enlarged and penetrated by leukaemic infiltrates (Fig. 9–11). By contrast, the spleen is generally unaffected. Haematologically, most of the leukoses are lymphoid; myeloid and reticulo-cellular types are less frequently encountered. Leukoses arose, as a rule, between 1 and 2 months after treatment. Subcutaneous injection of cellular infiltrates into newborns never induced local tumour formation: leukoses were the only type of tumour observed. The leukoses induced by cell-free extracts were easily transplantable into both young and adult hamsters by cellular grafts which produced large local tumours. Exactly the same picture of disease as in the case of cell-free filtrates from hamster skin tumours was observed when hamsters from the colony without spontaneous tumours were treated when newly born with twice-centrifuged and twice-filtered extracts from the cell-free-induced primary hamster leukaemias or its transplants (Table I, Fig. 4). In several cases when the twice-filtered nutrient fluid from tissue cultures from hamster skin tumours was given to newborn hamsters, leukaemias were induced. These were also first manifest in the liver (Table I).

Again, animals of our own colony with spontaneous tumours in general reacted negatively.

Centrifuged extracts from hamster skin tumours given to newborn Wistar rats resulted, in several cases—depending on the individual origin of the starting material (skin tumour)—in the formation of reticuloses and reticulum cell sarcomas (Table II). These usually started in the spleen and extended always to the liver

TABLE II.—*Reticuloses and Reticulum Cell Sarcomas in Rats by Induction of Centrifuged Extracts from Epithelial Hamster Skin Tumours*

| Number of litters | | Number of rats | Animals with reticuloses |
|---|---|---|---|
| First experiment: | 6 | 50 | 29 |
| Second experiment: | 5 | 41 | 27 |
| Total: | 11 | 91 | 56 = 61·5 per cent |

and sometimes to the lymph nodes and the thymus (Fig. 12–14). The enlarged spleen, often weighing 15 g., contained a large number of tumour nodules of various sizes (Fig. 12); the liver revealed macroscopically visible infiltrations (Fig. 12). These rat reticuloses had latency periods of 2 to 3 months. They were transplantable into younger Wistar rats in which they gave rise to local tumours. In many cases cell-free filtrates prepared from these rat reticuloses, when applied to newborn hamsters, produced nodular reticuloses or leukoses which started in the liver (Fig. 5; Table I). No positive results have so far been obtained by the introduction of cell-free extracts from hamster skin tumours and hamster leukaemias into newborn mice ($>$200 animals).

The electron microscopic investigation revealed the following results. The Papova virus (Fig. 2) which regularly occurs in hamster skin tumours was never seen in the leukoses and reticuloses from hamsters and rats. However, a small number of virus particles, similar in structure and size to leukaemia virus of mouse and chicken (Fig. 6–8) were seen in the hamster leukoses. The particles are 90 to 100 m$\mu$ in diameter and have an envelope and a mature or immature nucleoid of $\sim$55 to 60 m$\mu$ in diameter. The virus particles bud from membranes of the endoplasmic reticulum (Fig. 8). The formation of particles in the outer cell membrane as seen in the case of the murine leukaemia viruses has not so far been observed.

### DISCUSSION

The induction of hamster leukoses by cell-free extracts from hamster tumours indicates that in this species as in others certain, possibly all, leukaemias are of viral aetiology. However, there remains a certain obscurity concerning the nature and origin of the causative virus. The material from which the cell-free extracts were primarily obtained, the epithelial hamster skin tumours, contained masses of Papova virus (Fig. 2) whereas this virus has not previously been detected in leukoses induced by cell-free extracts. Instead, these leukoses contained another, completely different virus, which is quite similar to murine and chicken leukaemia viruses. We have so far not been able to demonstrate this virus electron microscopically in hamster skin tumours. On the basis of the fact that hamster leukoses can be transmitted to other animals by cell-free extracts we are inclined to conclude that this second, larger, virus, with an envelope, is the cause of hamster and rat leukoses and reticuloses in our experiments and that it is not the Papova virus. Thus one might assume that epithelial hamster skin tumours also contain a hamster leukaemia virus as passenger, resembling the way in which mouse leukaemias are frequently associated with another Papova virus, i.e. the polyoma virus. This assumption is also supported by the above mentioned spontaneous occurrence of skin tumours and lymphomas in our hamster colony as well as by the occasional presence of both tumour forms in the same animal.

Special mention must be made of the remarkable fact that leukoses were only induced in a high percentage of hamsters by cell-free extracts from hamster skin tumours or from hamster leukoses, if the animals were treated as newborns and if the animals were derived from a hamster colony *free* of spontaneous tumours. In contrast, the animals of our own colony in which both skin tumours and lymphomas occurred spontaneously were relatively insensitive to the filtrates. We believe that the animals of our hamster colony, on account of the general contamination with leukaemia virus, have acquired *via* the placenta or milk a relative immunity against the virus. This is perhaps the explanation of the fact that the attempts of Toth (1967) to transmit hamster lymphomas by cell-free extracts were negative.

Finally it is necessary to mention the interesting work of Greene and Harvey (1967) who obtained local subcutaneous lymphomas in adult hamsters after *transplantation* of different heterologous tumours; attempts at cell-free transmission were also negative. In this case, these authors suggest, that the lymphomas are caused by an immunological process and not by virus. With respect to our results with cell-free filtrates from homologous tumours an immunological mechanism causing the leukoses seemed to us very improbable.

---

### EXPLANATION OF PLATES

FIG. 1.—Histological picture of the epithelial skin tumour from Syrian hamster. H. & E. ×200.

FIG. 2.—Papova-virus in the nucleus from a cornified cell in an epithelial skin tumour of hamster. Electron microscopic picture of an ultra thin section. ×45,000.

FIG. 3.—Leukosis in a Syrian hamster induced by injection of cell-free filtrate from epithelial hamster skin tumours into the newborn animal. Latent period 7 weeks. Tumourous enlarged liver and thymus.

FIG. 4.—Leukosis in a Syrian hamster 6 weeks after introduction of cell-free filtrate from hamster leukosis. Note the enormously enlarged tumourous liver.

FIG. 5.—Leukosis in a Syrian hamster 7 weeks after the introduction of a cell-free filtrate from an induced reticulosis of the rat.

FIG. 6 and 7.—Electron microscopic pictures of virus particles in the cytoplasm of cells from cell-free filtrate-induced hamster leukaemias. Ultra thin sections ×60,000.

FIG. 8.—Budding of a virus particle at the membrane of the endoplasmic reticulum from an induced hamster leukaemia. ×60,000.

FIG. 9.—Infiltration of the liver in a cell-free filtrate-induced lymphatic hamster leukaemia. H. & E. ×150.

FIG. 10.—As Fig. 9, ×600.

FIG. 11.—Leukaemic infiltration into the muscle from lymphatic cell-free induced hamster leukaemia. H. & E. ×150.

FIG. 12.—Reticulosis in a rat 9 weeks after introduction of a subcellular extract from hamster skin tumour into the newborn animal. Enlarged liver and spleen with many white tumour nodules.

FIG. 13.—A rat treated similarly to that shown in Fig. 12. Besides the spleen, the thymus and lymph nodes in the neck and axillae are affected.

FIG. 14.—Histological picture of a reticulum cell sarcoma from the spleen of the rat treated with an extract from hamster skin tumour. H. & E. ×400.

1

2

Graffi, Schramm, Bender, Graffi, Horn and Bierwolf.

3

4

5

6

7

8

Graffi, Schramm, Bender, Graffi, Horn and Bierwolf.

9

10

11

Graffi, Schramm, Bender, Graffi, Horn and Bierwolf.

12

13

14

Graffi, Schramm, Bender, Graffi, Horn and Bierwolf.

SUMMARY

Cell-free filtrates from hamster skin tumours in which Papova virus could be detected electron microscopically were able, if introduced during the neonatal period, to induce leukoses, mainly lymphomas, in Syrian hamsters derived from a colony in which leukaemias did not occur spontaneously. Several hamster skin tumour extracts produced a high percentage of reticuloses and retothelial sarcomas also in Wistar rats. Hamster leukoses induced in this manner can be transmitted further to hamsters by application of cell-free filtrates to newborn animals. In these hamster leukoses a virus, morphologically similar to viruses which cause murine leukaemia has been found and has been seen budding from the membranes of the endoplasmic reticulum. Papova virus has not been encountered in these leukaemias. The reticuloses induced by hamster material in rats were often also re-transmissable to newborn hamsters by cell-free material.

### REFERENCES

Graffi, A., Schramm, T., Bender, E., Bierwolf, D., and Graffi, I.—(1967) *Arch. Geschwulstforsch*, **30**, 227.

Greene, H. S. N. and Harvey, E. K.—(1967) *Am. J. Path.*, **51**, 447.

Horn, K.-H. and Siewert, R.—(1968) *Acta biol. med. germ.*, **20**, 103.

Toth, B.—(1967) *Cancer Res.*, **27**, 1430.

ADDENDUM.

Filtered nutrient fluid from tissue cultures from hamster leukoses has also a strong leukaemogenic effect after application to newborn hamsters (40 leukoses/89 animals = 45 per cent). The cell-free nutrient fluid from tissue cultures from normal embryos of hamsters, rats and HeLa-cells inoculated with cell-free extracts from hamster leukoses or hamster skin tumours has the same effect (24 leukoses/54 animals = 44 per cent). Filtrates from normal organs of rats, guinea-pigs and hamsters and cell-free extracts of 6 different transplantable carcinomas and sarcomas of rats and mice and 4 different hamster sarcomas after inoculation to newborn hamsters (about 100 animals) yielded so far a negative result. Positive results were obtained by cell-free extracts from one intraperitoneally growing multiple human sarcoma from a young girl.